# Effects of slice orientation on reproducibility of sequential assessment of right ventricular volumes and ejection fraction: short-axis vs transverse SSFP cine cardiovascular magnetic resonance

**DOI:** 10.1186/s12968-016-0282-x

**Published:** 2016-09-22

**Authors:** Luigia D’Errico, Mariana M. Lamacie, Laura Jimenez Juan, Djeven Deva, Rachel M. Wald, Sebastian Ley, Kate Hanneman, Paaladinesh Thavendiranathan, Bernd J. Wintersperger

**Affiliations:** 1 Department of Medical Imaging, Peter Munk Cardiac Centre, University Health Network, Toronto, Canada; 2 Department of Medical Imaging, University of Toronto, Toronto, Canada; 3 Department of Medical Imaging, Sunnybrook Health Science Centre, Toronto, Canada; 4 Department of Medical Imaging, St. Michaels Hospital, Toronto, Canada; 5 Department of Medicine, University of Toronto, Toronto, Canada; 6 Department of Medical Imaging, Toronto General Hospital, 1 PMB-273, 585 University Avenue, M5G 2N2 Toronto, ON Canada

**Keywords:** Cardiovascular magnetic resonance, Cardiac output, Ventricles

## Abstract

**Background:**

Test-retest reproducibility is of utmost importance in follow-up of right ventricular (RV) volumes and function; optimal slice orientation though is not yet known. We compared test-retest reproducibility and intra-/inter-observer variability of right ventricular (RV) volumes and function assessed with short-axis and transverse cardiovascular magnetic resonance (CMR).

**Methods:**

Eighteen volunteers underwent cine CMR for RV assessment obtaining ventricular coverage in short-axis and transverse slice orientation. Additional 2D phase contrast flow imaging of the main pulmonary artery (MPA) was performed. After complete repositioning repeat acquisitions were performed. Data sets were contoured by two blinded observers. Statistical analysis included Student’s *t*-test, Bland-Altman plots, intra-class correlation coefficient (ICC) and 2-way ANOVA, SEM and minimal detectable difference calculations.

**Results:**

Heart rates (65.0 ± 7.4 vs. 67.6 ± 9.9 bpm; *P* = 0.1) and MPA flow (89.8 ± 16.6 vs. 87.2 ± 14.9 mL; *P* = 0.1) did not differ between imaging sessions. EDV and ESV demonstrated an inter-study bias of 0.4 %[−9.5 %,10.3 %] and 2.1 %[−12.3 %,16.4 %] for short-axis and 1.1 %[−7.3 %,9.4 %] and 0.8 %[−16.0 %,17.6 %] for transverse orientation, respectively. There was no significant interaction between imaging orientation and interstudy reproducibility (*p* = 0.395–0.824), intra-observer variability (*p* = 0.726–0.862) or inter-observer variability (*p* = 0.447–0.706) by 2-way ANOVA. Inter-observer agreement by ICC was greater for short axis versus transverse orientation for all parameters (0.769–0.986 vs. 0.625–0.983, respectively). Minimal detectable differences for short axis and transverse orientations were 10.1 mL/11.5 mL for EDV, 8.3 mL/8.4 mL for ESV and 4.1 % vs. 4.7 % for EF, respectively.

**Conclusion:**

Short-axis and transverse orientation both provide reliable and reproducible measures for follow-up of RV volumes and global function. Therefore, additional transverse SSFP cine CMR may not necessarily be required if performed for the sole purpose of quantitative volumetric RV assessment.

## Background

Accurate and reproducible quantification of right ventricular volumes and function plays a crucial role in the diagnosis of various cardiac diseases. It also guides clinical decision-making and monitoring of therapy in many conditions. These include pathologies primarily affecting the right ventricle (RV) such as arrhythmogenic right ventricular cardiomyopathy (ARVC), tricuspid regurgitation, pulmonary hypertension as well as congenital heart disease (CHD) entities such as Tetralogy of Fallot (ToF), Ebstein’s and shunt diseases [1–6].

Various studies have established cardiovascular magnetic resonance (CMR) as the standard of reference in assessment of RV dimensions and function [7–10]. Furthermore, accurate quantification of RV dimensions has gained attention as an important predictor of outcomes in heart failure and proven essential for therapy decisions and surveillance [5–8, 11, 12] Other than left ventricular assessment the complex anatomy of the RV imposes a challenge to other volumetric measurement methods with additional limitations of echocardiography related to limited acoustic windows [4, 13]. This may be further complicated by various RV morphologies in CHD.

While the acquisition of cine steady state free prcession (SSFP) data in short axis orientation (SAO) is the accepted standard for assessment of the left ventricle (LV) in CMR, both, transverse as well as short axis slice orientations have been applied for analysis of the RV. However, acquisition of an additional transverse stack for the sole purpose of RV volumetric assessment not only lengthens the total examination time and as such effects imaging workflow and capcities but also may negatively impact patients’ comfort and compliance.

Several studies have demonstrated that transverse cine orientation has better intra- and inter-observer reproducibility than SAO cine in patient populations with various anatomies [7, 14–17].

Additionally, longitudinal follow-up examinations using CMR and identification of changes and pattern of changes over time have gained attention especially in CHD [12, 18]. CMR has previously demonstrated high test-retest reproducibilities for the assessment of RV and LV volumes with superior results in comparison to echocardiography [19–21]. While few studies have aimed for the test-retest reproducibility with respect to LV and RV size and function using SAO slices [19, 22–25], no study has yet directly compared the influence of slice orientation on test-retest reproducibility for RV assessment. Therefore it still remains unclear which slice orientation provides better overall performance including test-retest reproducibility for longitudinal follow-up of RV volumes and ejection fraction (EF).

We hypothesize that SAO is not inferior to transverse orientation cine SSFP in respect to the overall variability of longitudinal comparison for RV volume and function assessment.

The aim of our study therefore was to evaluate test-retest variability of cine SSFP applied in both orientations approaches in addition to intra- and inter-observer variability in a cohort of healthy volunteers undergoing two serial CMR exams.

## Methods

### Study population

Twenty-one healthy volunteers aged 18 or older with no personal or family history of cardiovascular disease were recruited. The following exclusion criteria were applied: contraindication to MRI, claustrophobia, possible pregnancy in female candidates.

Data from three participants (2 male/1 female) were excluded from the study because of either the short axis or transverse cine SSFP data sets were missing or affected by substantial motion artifacts. The age of the final cohort of 18 participants (10 male/8 female) ranged from 22.2 to 45.6 years (33.0 ± 6.7 years).

### CMR

All CMR was performed at 1.5 T (MAGNETOM Espree, Siemens Healthcare GmbH, Erlangen, Germany) using body surface arrays for optimized signal reception. After localization of cardiac axes, ECG retrogated cine SSFP was applied for analysis of ventricular function. This included a stack of parallel short axis cine SSFP acquisitions with coverage from the atrio-ventricular plane to the apex with a slice thickness of 6 mm (2 mm gap) and an in-plane resolution of 1.35 × 1.35 mm^2^ (matrix 224). With the acquisition of 13 lines/segment and parallel acquisition using GRAPPA (*R* = 2) the temporal resolution was 40 ms. In addition, a transverse stack of cine SSFP slices provided coverage from the main pulmonary artery (MPA) to the infracardiac diaphragmatic surface. Identical parameters of spatial and temporal resolutions were applied as detailed above. Further details were: flip angle 75°, repetition time 3.1 ms, echo time 1.3 ms, bandwidth 930Hz/Px.

Additional phase contrast (PC) flow imaging was performed in the main pulmonary artery (MPA) serving as an independent standard of reference for the RV stroke volume. After multiplanar localization of the MPA for perpendicular slice prescription, an ECG retrogated through-plane velocity encoded PC spoiled gradient recalled echo (sGRE) technique measurement was performed in free-breathing with two averages. Spatial resolution was 1.56x1.56 mm^2^ (matrix 256) with a slice thickness of 5 mm. With the acquisition of 2 lines/segment the achieved temporal resolution was 26.9 ms. Further sequence details included: flip angle 30°, repetition time 13.5 ms, echo time 3.5 ms, bandwidth 391Hz/Px.

For both retrogated techniques, cine SSFP and PC flow measurements, the number of phases reconstructed per RR cycle was chosen to match the temporal distance between phases to the acquired temporal resolution.

In order to test for interstudy variability all above measurements were repeated after a 5–8 min biobreak with the volunteer getting off the table (at home position) and exiting the scan room. After the break the volunteers were repositioned on the table with acquisition of new localizers and re-planning/re-scanning of above described cine SSFP stacks and PC flow measurements.

### CMR data analysis

**Fig. 1:**
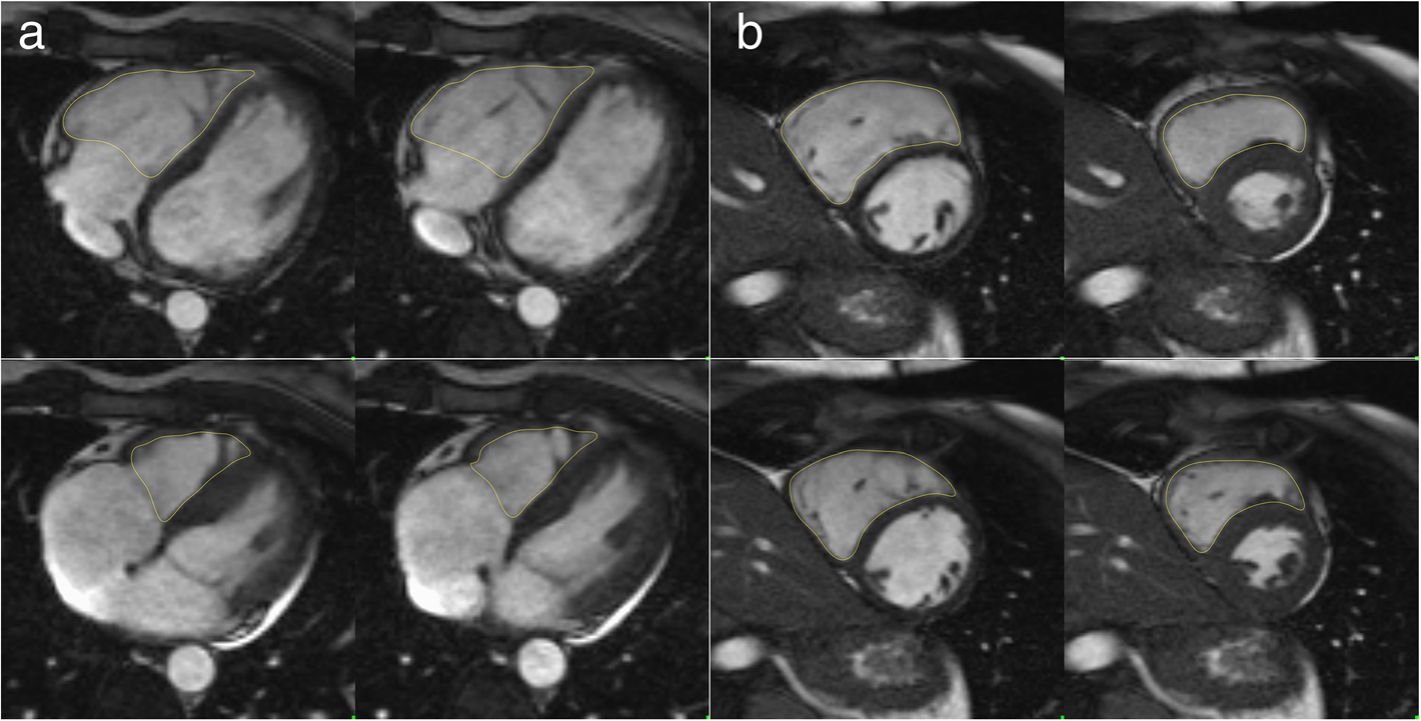
Display of RV end-diastolic (upper row) and end-systolic (lower row) endocardial contours in **a** transverse and **b** short axis orientation

Semi-automated segmentation of the RV volume in transverse and short axis cine SSFP data of all volunteers was performed by two observers (L.D., M.M.L.) using a commercially available post-processing software (QMass MR 7.6, Medis, Leiden, The Netherlands) (Fig. 1). Both observers have undergone a dedicated cardiac imaging fellowship training. Furthermore both observers contoured 10 non study related RV data sets to ensure identical approaches of RV contouring.

Anonymized image data was presented to observers in a random fashion in order to avoid back to back segmentation of transverse and short axis data sets in the same patient. First, end-diastolic and end-systolic frames were chosen for the RV segmentation followed by drawing of the endocardial RV contours at end-diastole and end-systole. Trabeculations were excluded from the mass and included in the RV blood pool. Following finalization of the RV contouring ventricular parameters, such as end-diastolic volume (EDV), end-systolic volume (ESV), stroke volume (SV) and ejection fraction (EF) were recorded. One observer (L.D.) repeated all contouring with a time interval of >10 days between both contouring sessions blinded to results of any previous contouring.

Available PC flow data sets for the MPA were analysed by a third observer (B.J.W.) blinded to results of RV volumetric measurements. Flow data quantification was performed using semiautomated postprocessing tools (syngo.MR Cardiac Flow, Siemens Healthcare GmbH, Erlangen, Germany). After initial automated contour seeding within the MPA and propagation across the entire series, contours were subsequently checked on all individual frames and modified whenever necessary. Furthermore, the stationary pixel based background correction was applied in order to correct for potential offsets. The forward volume, reverse volume and net forward volume per heartbeat were recorded for further analysis. In addition, this observer also visually evaluated the available cine SSFP data sets for possible tricuspid regurgitation which would affect the comparability of RV stoke volumes to the MPA forward flow volume.

For the purpose of analysis of potential heart rate variation between both scanning session, the average RR interval during the retrogated MPA PC flow acquisition was recorded.

### Statistical analysis

All continuous data is presented as mean ± standard deviation unless otherwise stated. Student’s *t*-test was applied for comparison of individual volumetric results as well as for comparison with recorded MPA flow data. Statistical analysis also included Bland-Altman analysis for the recorded RV volumetric and functional data, intra-class correlation coefficient (ICC) for inter-observer variability analysis and 2-way ANOVA to assess for the effect of slice orientation on interstudy as well as intra-/inter-observer variability of volumetric RV results.

In addition, the standard error of the measurement (SEM) for EF and each volumetric parameter was evaluated by calculating the square root of the mean square error for each slice orientation performed based on one-way ANOVA. In this analysis the parameter or interest was used as the dependent variable, while patient ID was used as fixed factors. Subsequently the minimal required change between two separate examination time points required for each parameter beyond which a real change could be assumed by a chosen cine MRI slice orientation (short axis, transverse) was calculated as 2x SEM [26]. A standard cut-off for significance was applied with *p* < 0.05 with Bonferroni corrections applied for multiple repeated testing when required.

## Results

Evaluation of RV volumes and function based on cine SSFP was successful in the entire study cohort of 18 healthy volunteers with a total of 36 cine SSFP volume stacks to be contoured (18 transverse, 18 short axis). In all but one volunteer MPA PC flow assessment was successfully analysed for both sessions. A single volunteer demonstrated a significant variation in RR interval (1039 ± 422 ms) in one session of the MPA PC flow data related to transient arrhythmia making this flow measurement unreliable as a reference for the RV output. This volunteer’s MPA flow data was therefore not used for any comparison of flow related data between both measurement sessions. All other flow and volumetric data of this individual were not affected by any arrhythmia and as such included in respective subsequent data analysis.

In the 17 volunteers where reliable heart rate recording was available, no significant differences between the recorded heart rates between scan session 1 and 2 was observed (65.5 ± 7.4 bpm vs. 67.3 ± 10.2 bpm: *P* = 0.12).

### Main pulmonary artery flow and RV stroke volume

**Fig. 2:**
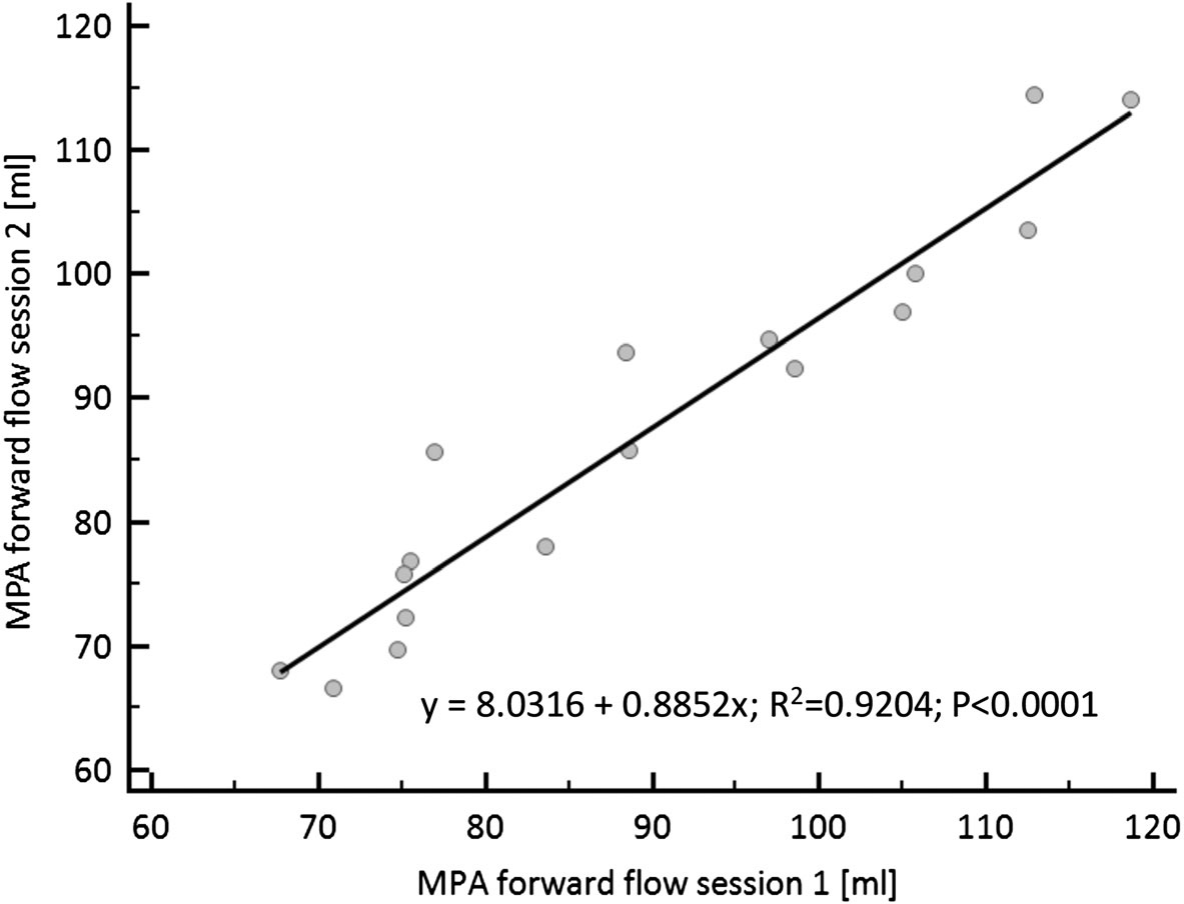
Regression analysis of acquired MPA flow data in session 1 (x-axis) and session 2 after repositioning (y-axis)

Quantification of the MPA flow for measurement sessions 1 and 2 demonstrated strong correlation between both sessions (*r* = 0.959; *P* < 0.001) without any significant difference in forward flow (Table 1) (Fig. 2). The resulting RV output per minute averaged to 5948.2 ± 1189.1 ml/min (3876.0–7936.1 ml) for session 1 and 5717.5 ± 1098.7 ml/min (3842.4–7617.6 ml) for session 2 (*P* = 0.1).

**Table 1:** Overview of RV Stroke Volume (SV) results displayed for study, observer and slice orientation in comparison to main pulmonary artery (MPA) flow volumes. Data presented per scan is based on the number of available MPA PC flow reference data without trigger errors (Scan 1/*n* = 17; Scan 2/*n* = 18; see Methods for details)

	Observer 1		Observer 2		Observer 3
	Transverse	Short axis	Transverse	Short axis	MPA PC flow
Scan 1 (*n* = 17)	92.5 ± 15.8 (66.9–123.3)	86.9 ± 14.5 (64.0–114.6)	92.0 ± 15.4 (60.1–121.2)	84.9 ± 15.6 (56.9–114.5)	89.8 ± 16.6 (67.7–118.7)
Scan 2 (*n* = 18)	90.3 ± 14.1 (68.3–116.8)	87.1 ± 15.1 (61.3–116.3)	89.2 ± 14.2 (60.6–114.0)	84.9 ± 13.7 (61.8–111.3)	87.2 ± 14.9 (66.6–114.4)

All data in [*ml]*; data is presented in mean ± standard deviation; range in parentheses

No significant differences were observed for RV SV results of both observers and both imaging orientations in comparison to the respective MPA forward volumes as measured by PC flow measurements (all *P* > 0.05) (Table 1). In addition RV SV results of both observers demonstrated strong positive correlation to independently evaluated MPA flow data for transverse orientation (observer 1: *r* = 0.896; observer 2: *r* = 0.873; all *P* < 0.001) and SAO (observer 1: *r* = 0.826; observer 2: *r* = 0.824; all *P* < 0.001). For both observers the transverse orientation RV SV strongly correlated to SAO derived RV SV data (observer 1: *r* = 0.845; observer 2: *r* = 0.924; all *P* < 0.001) without significant differences in pairwise comparison (all *P* > 0.05) (Table 2)

**Table 2:** Overview of RV volumetric results displayed for study, observer and slice orientation

	Scan session 1				Scan session 2			
	Observer 1		Observer 2		Observer 1		Observer 2	
	Transverse	Short axis	Transverse	Short axis	Transverse	Short axis	Transverse	Short axis
EDV (ml)	163.2 ± 26.5 (117.2–212.6)	160.5 ± 26.2 (117.7–215.1)	164.9 ± 25.5 (116.3–215.9)	157.8 ± 27.6 (106.5–215.5)	161.4 ± 25.9 (112.3–220.0)	160.2 ± 28.5 (109.1–223.1)	163.0 ± 26.8 (113.2–224.0)	157.3 ± 25.8 (111.6–214.4)
ESV (ml)	71.3 ± 13.6 (49.3–96.5)	74.4 ± 14.7 (46.3–105.9)	73.5 ± 13.0 (56.2–104.5)	73.6 ± 14.3 (47.5–105.5)	71.2 ± 15.4 (43.9–103.1)	73.1 ± 15.6 (45.6–107.1)	73.9 ± 14.9 (50.1–110.0)	72.3 ± 14.3 (49.8–103.2
SV (ml)	91.9 ± 15.6 (66.9–123.3)	86.0 ± 14.6 (64.0–114.6)	91.4 ± 15.1(60.1–121.2)	84.1 ± 15.4 (56.9–114.5)	90.3 ± 14.1 (68.3–116.8)	87.1 ± 15.1 (61.3–116.3)	89.2 ± 14.2 (60.6–114.0)	84.9 ± 13.7 (61.8–111.3)
EF (%)	56.4 ± 3.5 (49.9–60.4)	53.8 ± 4.1 (47.3–60.9)	55.4 ± 3.5 (50.2–62.9)	53.4 ± 3.4 (46.6–60.3)	56.1 ± 4.9 (47.8–64.5)	54.5 ± 3.8 (47.8–60.9)	54.8 ± 3.7 (47.5–61.4)	54.1 ± 3.7 (46.0–60.2)

Data is presented in mean ± standard deviation; range in parentheses * *P* < 0.05

For inter-observer agreement the ICC demonstrated higher values in short-axis vs. transverse orientation for SV measures (0.954 [0.882, 0.983] vs. 0.936 [0.837, 0.976]) (Table 3).

**Table 3:** Intra-class correlation coefficient (ICC) results for analysis of inter-observer variability

	Transverse	Short axis
RV EDV	0.983 (0.954, 0.993)	0.986 (0.963, 0.995)
RV ESV	0.926 (0.814, 0.972)	0.955 (0.883, 0.983)
RV SV	0.936 (0.837, 0.976)	0.954 (0.882, 0.983)
RV EF	0.625 (0.238, 0.841)	0.769 (0.482, 0.907)

ICC values with 95 % CI in parentheses

No significant interaction between imaging orientation was demonstrated for interstudy (*F* = 0.402, *p* = 0.527), inter-observer (*F* = 0.143, *p* = 0.706) and intra-observer (*F* = 0.095, *p* = 0.759) variability of SV results.

### End-diastolic volumes

**Fig. 3:**
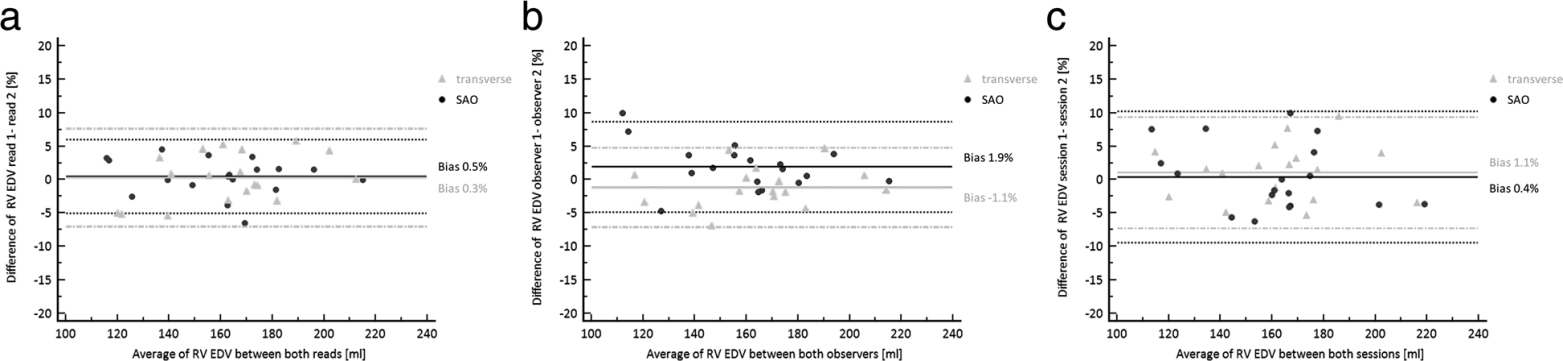
Bland-Altman analysis of EDV results of both slice orientations for **a** intra-observer variation, **b** inter-observer variation and **c** interstudy (session) variation

Contouring of both observers did not demonstrate any significant differences between short axis and transverse cine SSFP for EDV (Table 2). EDV results based on SAO demonstrated strong correlation to those based on transverse slice orientation (observer 1: *r* = 0.952; observer 2: *r* = 0.982; all *P* < 0.001). Results for EDV demonstrated a bias of 0.4 % (−9.5 %,10.3 %) for short axis and 1.1 % (−7.3 %,9.4 %) for transverse orientation between both sessions (Table 4) (Fig. 3). In regard to inter- and intra-observer variability SAO demonstrated a bias of 1.9 % (−4.8 %, 8.7 %) and 0.5 % (−5.1 %, 6.0 %) while for transverse slice orientation the bias was −1.1 % (−7.1 %, 4.8 %) and 0.3 %, 7.7 %), respectively (Table 4) (Fig. 3). For inter-observer agreement the ICC demonstrated minimally higher values in short-axis vs. transverse orientation for EDV (0.986 [0.963,0.995] vs. 0.983 [0.954,0.993]) but overlapping confidence intervals (Table 3).

**Table 4:** Bias and limits of agreement for interstudy, inter-observer and intra-observer variability for both slice orientations based on Bland-Altman analysis

	Transverse	Short axis
EDV		
Interstudy	1.1 (−7.3, 9.4)	0.4 (−9.5, 10.3)
Inter-observer	−1.1 (−7.1, 4.8)	1.9 (−4.8, 8.7)
Intra-observer	0.3 (−7.0, 7.7)	0.5 (−5.1, 6.0)
ESV		
Interstudy	0.8 (−16.0, 17.6)	2.1 (−12.3, 16.4)
Inter-observer	−3.2 (−17.6, 11.2)	1.0 (−11.2, 13.2)
Intra-observer	−1.4 (−14.5, 11.7)	−0.4 (−12.4, 11.6)
EF		
Interstudy	0.6 (−11.2, 12.4)	−1.5 (−7.2, 10.2)
Inter-observer	1.6 (−9.1, 12.4)	0.5 (−8.7, 9.7)
Intra-observer	1.6 (−7.2, 10.5)	0.7 (−8.4, 9.7)

Values demonstrate % bias with BA limits of agreement in parentheses

2-way ANOVA did not demonstrate significant interaction between imaging orientation and interstudy (*F* = 0.050; *p* = 0.824) as well as inter- (*F* = 0.382; *p* = 0.537) and intra-observer (*F* = 0.123; *p* = 0.726) variability for EDV results.

### End-systolic volumes

**Fig. 4:**
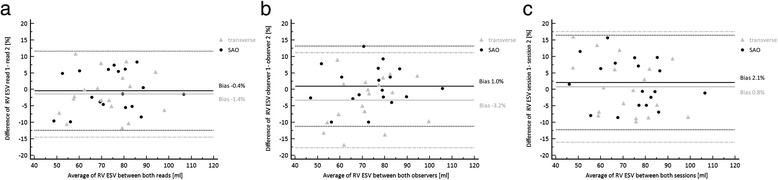
Bland-Altman analysis of ESV results of both slice orientations for **a** intra-observer variation, **b** inter-observer variation and **c** interstudy (session) variation

No significant differences were identified with respect to ESV data for both observers between short axis and transverse cine SSFP orientation (Table 2). ESV results based on SAO demonstrated strong correlation to those based on transverse slice orientation (observer 1: *r* = 0.925; observer 2: *r* = 0.904; all *P* < 0.001). Bland-Altman analysis revealed a bias of 2.1 % (−12.3 %, 16.4 %) and 0.8 % (−16.0 %, 17.6 %) between both studies for short axis and transverse orientation, respectively (Table 4) (Fig. 4). In regard to inter- and intra-observer variability of ESV data SAO demonstrated a bias of 1.0 % (−11.2 %, 13.2 %) and −0.4 % (−12.4 %, 11.6 %) while for transverse slice orientation the bias was −3.2 % (−17.6 %, 11.2 %) and −1.4 % (−14.5 %, 11.7 %), respectively (Table 4) (Fig. 4). For inter-observer agreement the ICC also demonstrated higher values in short-axis vs. transverse orientation for ESV (0.955 [0.883, 0.983] vs. 0.926 [0.814, 0.972]) with again overlapping confidence intervals (Table 3).

No significant interaction between imaging orientation was demonstrated for interstudy (*F* = 0.069, *p* = 0.792), inter-observer (*F* = 0.581, *p* = 0.447) and intra-observer (*F* = 0.119, *p* = 0.730) variability of ESV results.

### Ejection fraction

**Fig. 5:**
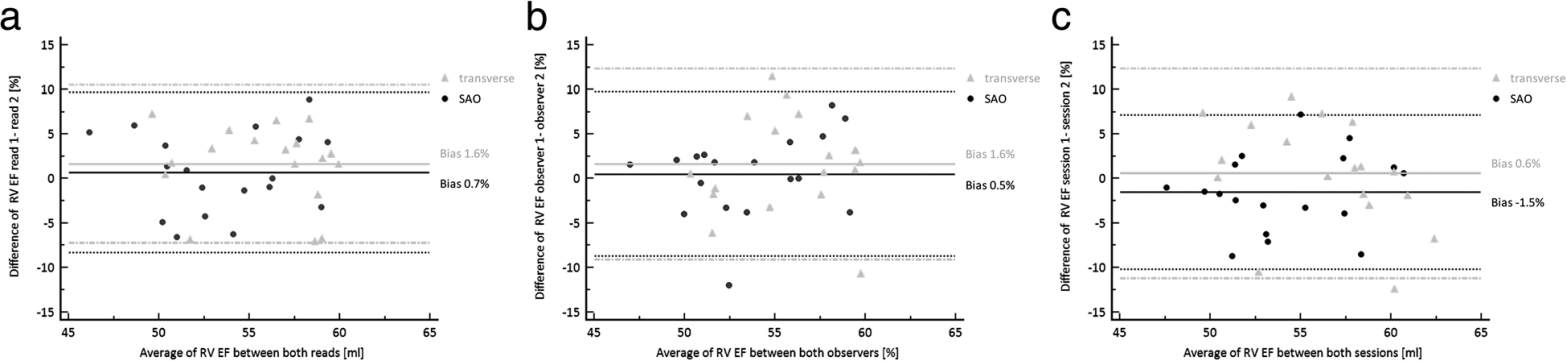
Bland-Altman analysis of EF results of both slice orientations for **a** intra-observer variation, **b** inter-observer variation and **c** interstudy (session) variation

Resulting RV EF did not demonstate any significant differences between short axis and transverse cine SSFP orientation for the two observers (Table 2). The inter-observer agreement for RV ejection fraction as assessed by ICC was lower than for individually measured volumes (e.g., EDV, ESV) (Table 3). It was again higher for SAO (0.769 [0.482, 0.907]) than for transverse orientation (0.625 [0.238, 0.841] (Table 3). The bias for EF measurements between both studies as assessed by Bland-Altman plots was −1.5 % (−7.2 %, 10.2 %) for a stack of short axis slices in comparison to 0.6 % (−11.2 %, 12.4 %) when using a stack of transverse cine SSFP slices for RV volumetric evaluation (Table 4) (Fig. 5). For inter-observer variability the bias was 0.5 % (−8.7 %, 9.7 %) for short axis and 1.6 % (−9.1 %, 12.4 %) for transverse orientation and for intra-observer variability the bias was 0.7 % (−8.4 %, 9.7 %) and 1.6 % (−7.2 %, 10.5 %), respectively (Table 4) (Fig. 5).

Again, no significant interaction between imaging orientation was demonstrated for interstudy (*F* = 0.726, *p* = 0.395), inter-observer (*F* = 0.338, *p* = 0.561) and intra-observer (*F* = 0.031, *p* = 0.862) variability of ESV results.

### Test retest reproducibility

For all assessed parameters SAO of cine SSFP demonstrated slightly better test-retest reproducibility than transverse slice orientation. Minimal detectable differences for short axis and transverse orientations were 10.1 ml vs. 11.5 ml for EDV and 8.3 ml vs. 8.4 ml for ESV. For RV SV the minimal detectable differences were 8.3 ml vs. 10.0 ml with short axis and transverse cine SSFP orientations respectively, while the minimal detectable difference for global systolic RV function as demonstrated by EF was 4.1 % for a short axis slice orientation and 4.7 % for a transverse slice orientation.

## Discussion

This study demonstrates that there is no significant effect of the slice orientation on accuracy or intra-observer and inter-observer variability of volumetric and functional assessment of the right ventricle in a cohort with normal RV morphology and size. Furthermore, it establishes that in this cohort there is no significant difference in test-retest-reproducibility between both slice orientations. While previous studies have focused on the effects of slice orientation on intra- and interobserver variability in RV volumetric analysis, to the best of our knowledge this study presents the first report on test-retest reproducibility comparison of short axis and transverse slice orientations cine CMR based RV volumetric assessment.

While for the acquisition of RV volumetric data either transverse or short axis slice orientations have been recommended for cine SSFP [27], published normal values for RV size and function are almost exclusively based on SAO and allow further subcategorization for gender and age [14, 28–31]. As such there is limited available published normal data of RV volumetry on transverse slice orientation.

Various investigators have demonstrated significantly lower intra- and inter-observer variability for RV volume assessment based on transverse over short axis orientation for cine SSFP examinations. Alfakih et al. demonstrated better intra- and inter-observer reproducibility for transverse orientation in healthy volunteers, while Fratz et al. demonstrated significantly lower intra- and inter-observer variance for RV (and LV) volumetry utilizing transverse cine SSFP orientation in a population of repaired tetralogy of Fallot (rToF) patients [16]. Similar results have been demonstrated in patients with atrial redirection surgery for complete transposition of the great arteries as well as in patients with Ebstein anomaly [17, 32]. Other studies showed a similar trend but no clinical significance of findings. Clarke et al. categorized differences in intra- and inter-observer variability between both orientations as not clinically relevant [14, 15]. Applying PC flow measurements as an independent standard in our population, the results of this study confirms that slice orientation has no significant impact on either intra- nor inter-observer variability in assessment of RV volumes and global systolic function (Tables 2 and 4).

In addition to intra- and inter-observer variability few studies have also demonstrated differences in ventricular volumes between both slice orientations. In the work by Alfakih et al. short axis slice orientations resulted in significantly larger results for RV EDV (4.7 % difference) and RV ESV (10.7 % difference) while no differences were found for SV [7]. With 2.9 % lower RV EDV values and 5.8 % lower RV ESV values derived from short axis slice orientation Sarikouch et al. reported lower off-sets in healthy children and adolescents (8-20y); again no relevant relative differences were demonstrated for resulting SV (1.1 % lower) and EF (1.8 % higher) using short axis orinentation [33]. Fratz et al. demonstrated significant differences in volumes for both approaches in rToF patients, while Clarke et al. showed a small, though statistically significant difference between both slice orientations for RV ESV but no significant differences for overall RV size as measured by EDV in various types of CHD (excluding subaortic RV) [14, 16]. Results published by James et al. in fact demonstrated minimaly lower RV EDV when applying short axis slice orientation with no significant EF differences; in addition they applied PC flow measurements in the MPA as an independent standard of reference and demonstrated no differences in RV SV offsets to PC data [15]. With previous studies demonstrating inconsistent results with either overestimation or underestimation of RV size by short axis cine SSFP, our study confirms that with regard to RV volumes no differences exist in both orientations. While underlying reasons for differences in previous studies are hard to evaluate, inconsistency in contouring approaches might have played a role. In addition, transverse orientation cine add extra examination time and are more vulnerable to errors introduced by inconsistent breath-holding than SAO cines (related to the parallel orientation of the inferior RV boundaries to the slice orientation). In unwell patients longer scans result in greater fatigue and may increase inconsistency; as such this is more likely to result in physiological changes possibly resulting in misinterpretation as significant volumetric findings (e.g., differences in heart rate during acquisitions for both orientations may be misinterpreted as discrepancy between RV and LV stroke volumes) or may introduce errors into other calculations (e.g., volume assessment of valvular heart disease or intracardiac shunts).

In therapy guidance and longitudinal follow-up of various cardiac pathologies that affect ventricular volumes and function a high test-retest reproducibility is of utmost importance. For example, various investigators have proposed cut-off values for timing of pulmonary valve replacement (PVR) in patients with repaired tetralogy of Fallot and chronic pulmonary valve regurgitation. With an RV EDV of <160–165 ml/m^2^ or an RV ESV of <80–85 ml/m^2^ a normalization of RV size after PVR has been demonstrated [3, 5, 6]. Grotheus et al. have demonstrated the substantial impact of the high test-retest reproducibility of CMR (compared to echocardiography) in assessment of LV volumes and function with a much more sensitive identification of true changes in ventricular size and performance [25]. Few studies have also assessed interstudy reproducibility for RV parameters. Early investigations by Pattynama et al. with 40 consecutive scans in 2 volunteers over 6 weeks have demonstrated a large variability of results from scan to scan using SAO [23]. However, the applied techniques (spin-echo and free-breathing spoiled gradient echo) are no longer in use for cardiac functional imaging and as such the presented data does likely not reflect current imaging approaches [31]. Using breath-hold FLASH techniques in SAO Grothues et al. demonstrated low interstudy variability of RV parameters healthy subjects, patients with heart failure, and patients with LV hypertrophy [19]. They reported an RV interstudy reproducibility (range between groups) of 6.2 % (4.2 %–7.8 %) for EDV and 14.1 % (8.1 %–18.1 %) for ESV [19]. Most recently Blalock et al. again investigated a population with rTOF demonstrating a repeatability coefficient (2 SD of the differences; percent value of population mean) of 9 % for RV EDV and 13 % for RV ESV when the same observer contoured both examinations [24]. While no change in results was demonstrated for RV EDV when a second obsverver contoured the repeat study, the repeatability coefficient for RV ESV increased to 20 % [24]. While all above referenced studies on test-retest performance for RV size and function focused on SAO, no data is available for either transverse slice selection nor for a comparison of both approaches. Interpretation of our data confirms a high test-retest reproducibility of CMR in assessment of RV volumes and size which is independent of the slice orientation chosen for ventricular coverage. Calculation of the minimal detectable difference between consecutive examinations based on standard error of the measurement (SEM) computation demonstrate that for RV EDV both orientations can identify real changes as small as ~10-12 ml, while for RV EDV identifiable changes are even smaller with ~8–9 ml between studies. This data results in identification of real interstudy RV EF changes of as small as ~4–5 %.

Despite the fact that this study provides the first direct comparison of test-retest reproducibility between transverse and short axis slice orientation for RV volumes and function, the following limitations apply.

The study is composed on a relatively small number of individuals included, however as opposed to previous test-retest investigations we included MPA PC flow measurements as an independent reference standard. Furthermore, our study only recruited cooperative healthy volunteers without relevant arrhythmia and a high level of compliance to scanning instructions. In a symptomatic patient population the test-retest reproducibility therefore might be lower than reported above.

## Conclusion

This first report on direct comparison of short axis and transverse cine SSFP slice orientation with respect to test-retest reproducibility of RV volumes and function demonstrate equally high performance of both orientations. While there were comparable levels of intra- and interobserver variability and similar volumetric results of both slice orientations, volumetric analysis of transverse slice orientation cines did not demonstrate a significant advantage over short axis ventricular coverage. With standard coverage using SAO for LV assessment, this single data set also provides adequate assessment of RV size and function. As such, transverse cine SSFP imaging may be omitted for the sole purpose of RV volumetric assessment resulting in shortened scan protocols for improved workflow and patient comfort.

However, assessment of complex vascular and cardiac anatomy may still require the application of transverse cine SSFP as a non-oblique and reproducible orientation for insight into spatial relationships.

## Abbreviations

ANOVA: Analysis of variance
ARVC: Arrhythmogenic right ventricular cardiomyopathy
CHD: Congenital heart disease
CMR: Cardiovascular magnetic resonance
ECG: Electrocardiogram
EDV: End-diastolic volume
EF: Ejection fraction
ESV: End-systolic volume
FLASH: Fast low angle shot
GRAPPA: Generalized autocalibrating partial parallel acquisition
ICC: Intra-class correlation coefficient
LV: Left ventricle
MPA: Main pulmonary artery
MRI: Magnetic resonance imaging
PC: Phase contrast
PVR: Pulmonary valve replacement
rToF: Repaired Tetrology of Fallot
RV: Right ventricle
SAO: Short axis oblique
SD: Standard deviation
SEM: Standard error of the measurement
sGRE: Spoiled gradient recalled echo
SSFP: Steady state free precession
SV: Stroke volume
ToF: Tetralogy of Fallot
